# 3D Binder-free MoSe_2_ Nanosheets/Carbon Cloth Electrodes for Efficient and Stable Hydrogen Evolution Prepared by Simple Electrophoresis Deposition Strategy

**DOI:** 10.1038/srep22516

**Published:** 2016-03-07

**Authors:** Yundan Liu, Long Ren, Zhen Zhang, Xiang Qi, Hongxing Li, Jianxin Zhong

**Affiliations:** 1 Hunan Key Laboratory of Micro-Nano Energy Materials and Devices, Xiangtan University, Hunan 411105, PR China; 2 Laboratory for Quantum Engineering and Micro-Nano Energy Technology and School of Physics and Optoelectronics, Xiangtan University, Hunan 411105, PR China; 3 Institute for Superconducting and Electronic Materials, Australian Institute for Innovative Materials, University of Wollongong, Innovation Campus, North Wollongong, New South Wales 2500, Australia

## Abstract

We successfully developed a simple electrophoretic deposition (EPD) method to decorate the MoSe_2_ nanosheets on the carbon fiber surface of carbon cloth (MoSe_2_/CC). With this process, MoSe_2_ nanosheets can be uniformly and tightly deposited on this flexible conductor to form a 3D binder-free electrode for hydrogen evolution reaction (HER). The film thickness can also be controlled by the EPD time. Directly used as binder-free electrodes for hydrogen evolution reaction, the as-prepared 3D MoSe_2_/CC samples exhibit excellent catalytic activity in an acidic electrolyte (21 mA/cm^2^ at an over-potential of 250 mV). Variation of MoSe_2_ nanosheets film thickness in the electrodes could affect the catalytic activity, and it was found that the MoSe_2_/CC sample prepared with 60 min EPD time shows the highest HER activity amongst these different thickness samples. Moreover, stability tests though long-term potential cycles (no degradation after 1000 continuous potential cycles) and extended electrolysis confirm the exceptional durability of the catalyst. This development offers us an attractive and active 3D electrode for electrochemical water splitting.

As one of the most promising alternative power sources to fossil fuels, hydrogen produced from water splitting has attracted growing attention due to its highest energy density^1^,^2^,^3^. The most effective electro-catalyst for the hydrogen evolution reaction (HER) in an acidic media is Pt group metals, but their high cost and scarcity limit their commercial application^4^,^5^. Therefore, exploring inexpensive alternatives with high electro-catalytic activity and stability to replace Pt is highly desirable for sustainable development^6^. Recently, layered transitional-metal dichalcogenides (LTMDs) MX_2_, where M represents a transition metal and X represents S, Se or Te, have inspired tremendous research interest as inorganic electro-catalysts for HER due to their low-cost and electrochemical stability in acid^7^,^8^,^9^,^10^,^11^. In particular, theoretical and experimental investigations suggest that the exposed edges of MoS_2_ sheets are the active sites for hydrogen adsorption with a binding free energy of adsorbed H close to that of H_2_ gas and with the H coverage of about 50%^7^,^12^,^13^,^14^,^15^. Many methods of preparing catalytically active MoS_2_ nanosheets have been developed, but in contrast, relatively few methods of preparing catalytically active MoSe_2_ which shares similar crystal structures with MoS_2_^16^,^17^,^18^,^19^. The density functional theory calculations show prove that the Gibbs free energy for atomic hydrogen adsorption on MoSe_2_ edges is much close to thermo-neutral, and it was also reported that MoSe_2_ possesses a higher H coverage than MoS_2_, which means MoSe_2_ can be a novel HER catalyst exhibiting comparable or even higher catalytic activity to MoS_2_^16^,^20^.

In view of edges of these LTMDs with dangling bonds are catalytically active while the basal planes are electrochemically inert in HER, nano-sizing LTMDs, especially preparing ultrathin nanosheets becomes the primary strategy to increase the number of catalytically active edge sites for HER^9^,^13^. Unfortunately, besides synthesizing LTMDs nanosheets with high density of active edge sites, the catalytic activities of such TMD nanosheets for HER are still limited the construction of electrodes^21^. Prior to electrochemical measurements, most of HER catalysts are required to be effectively immobilized on conductive substrates^22^. For most studies of LTMDs electro-catalysts, the catalyst was firstly dropped on the surface of glass carbon (GC) electrode, and then the binder-Nafion or PTFE (polytetrafluoroethene) was spread over the surface to immobilize the catalyst^11^,^23^. However, such polymer binder would increase the series resistance and may block active sites and inhibit diffusion, leading to reduced catalytic activity. Such issues can be solved by several routes, such as the direct growth of the active phases on conductive substrates via chemical vapor deposition (CVD), or hydrothermal process^24^,^25^,^26^. Both CVD and hydrothermal process were effective for the deposition, however, the use of high temperature and excessive chemical treatments impose serious limitations on the practical applications.

The electrophoresis deposition (EPD) is a versatile technique to fabricate thin and thick coatings on electrically conductive substrates^27^. Most of the nano-objects have surface charges when they are dispersed in a solution, this surface charge can provide a highly dispersed solution^28^. When apply an electric field to the solution, these charged units in aqueous or non-aqueous suspension can move onto a shaped electrode of opposite charge under the role of applied electric field^29^. This method is fast, homogeneous, suitable for mass production and allows flexibility in the electrode shapes without binders. Due to its advantages, EPD has been widely investigated on its potential application for structural and functional coatings, laminar composites and fuel cell^30^,^31^,^32^. But the investigation of this method in preparation of LTMDs catalytic electrodes for HER is still limited^33^.

In this work, we successfully deposit uniform MoSe_2_ nanosheets on carbon cloth (denoted as MoSe_2_/CC) via an electrophoretic deposition process for using as an efficient flexible binder-free electrode in HER devices. Here the CC is used as a conductive support which is a cheap textile with high conductivity and excellent flexibility and strength. The use of CC as support for catalysts also facilitates the integration of the electrodes directly into devices for applications^21^,^34^,^35^,^36^. With the help of EPD, uniform MoSe_2_ nanosheets would be deposited in CC to form a MoSe_2_/CC electrode. Moreover, the thickness of the MoSe_2_ film can be easily controlled by the EPD time. Thanks to the nanostructured films composed of sheet-like structure and 3D network of CC, resulting in a large number of exposed active edges and superior electrical contact with current collector as well as flexibility, the well-designed 3D electrode exhibits excellent catalytic activity and durability in acidic media.

## Results and Discussions

**Figure 1:**
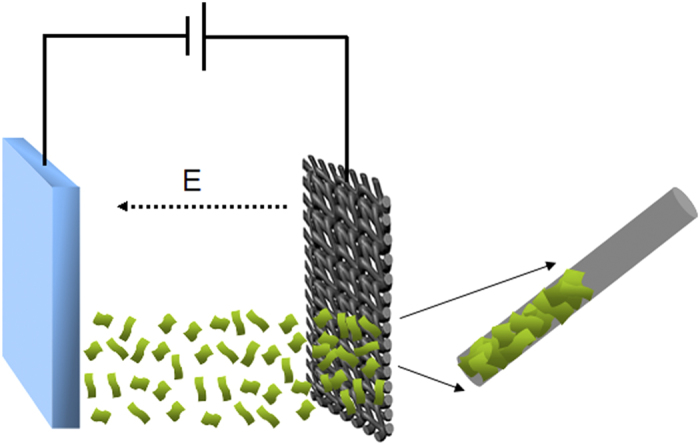
Schematic illustration for the electrophoresis deposition process of MoSe_2_ nanosheets on carbon cloth.

The method of EPD basically allows the formation of deposits from suspensions of charged particles, and a well-dispersed stable suspension will provide a better deposit. The MoSe_2_ nanosheets can be obtained using a facile hydrothermal exfoliation route reported in our previous work^37^. Moreover, these ultra-thin MoSe_2_ nanosheets are negatively charged and well-dispersed in aqueous solution due to the presence of carboxylic groups on their 2D surface. Figure 1 illustrates the EPD process of depositing MoSe_2_ nanosheets on CC. A carbon cloth and a stainless steel plate as two opposite electrodes were vertically oriented and separated by 1 cm in a beaker containing the MoSe_2_ nanosheets dispersion. A voltage of 30 V was applied between the CC (negative) and the metal counter electrode for different times. After that, the samples were dried at room temperature.

**Figure 2:**
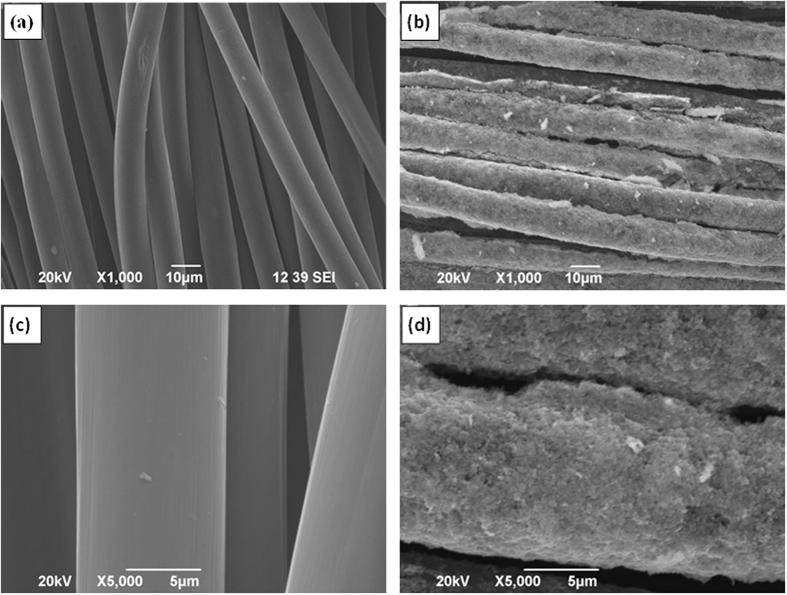
SEM images of (**a**), (**c**) pure carbon cloth and (**b**), (**d**) MoSe_2_ nanosheets/carbon cloth prepared with 60 min EPD time.

**Figure 3:**
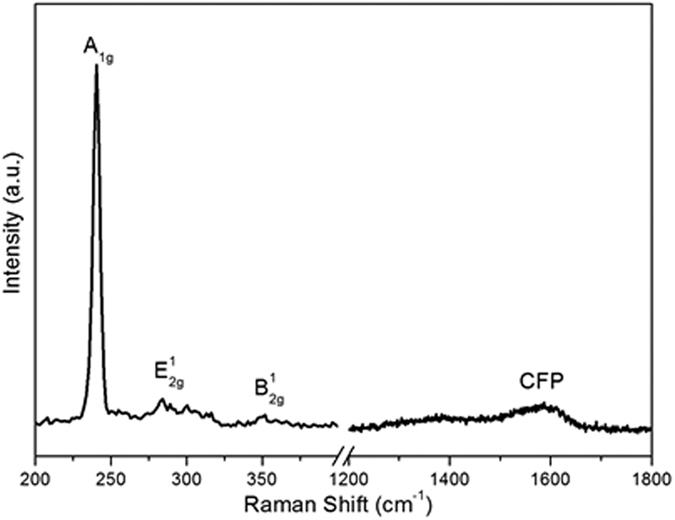
Raman spectra of MoSe_2_ nanosheets/carbon cloth prepared with 60 min EPD time.

**Figure 4:**
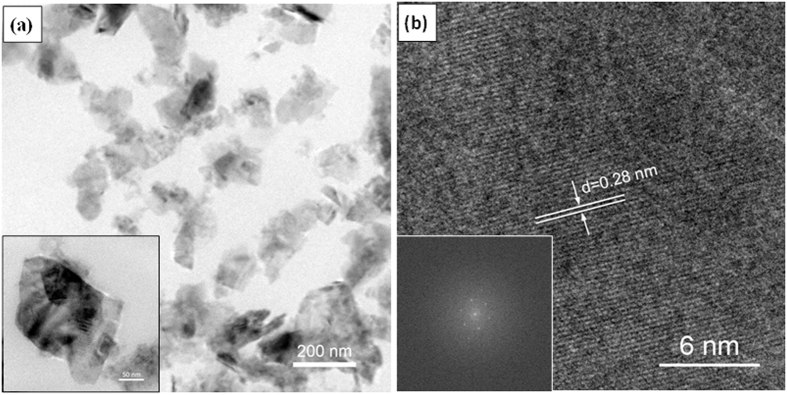
(**a**) TEM and (**b**) HRTEM images of exfoliated MoSe_2_ nanosheets.

The microstructure characterization of the CC before and after EPD process was performed by Scanning electron microscopic (SEM). As shown in Fig. 2(a,c), the fiber surface of CC is smooth before the EPD process. Interestingly, after 60 min EPD process, the surface of the CC became rough and decorated with nanosheets (Fig. 2(b,d)) which reveals the uniform distribution of MoSe_2_ nanosheets on each individual fiber of CC. Additional structural characterizations were acquired by X-ray diffraction (XRD) and Raman spectroscopy. Figure S1 (see the supporting information) shows the X-ray diffraction (XRD) pattern of the as-prepared MoSe_2_ nanosheets. The MoSe_2_ nanosheets prepared by the hydrothermal exfoliation displays a similar crystalline structure to that of pristine MoSe_2_, which can be indexed to the standard MoSe_2_ nanocrystal with a hexagonal structure (JCPDS: 87–2419). Figure 3 shows the Raman spectrum of the MoSe_2_/CC sample. Two apparent Raman characteristic bands at 243 cm^−1^ and 289 cm^−1^ for the MoSe_2_ can be observed, which correspond to the A_1g_ and E^1^_2g_ modes respectively^21^. In contrast with the Raman spectra of pure CC (see supporting information, Fig. S2), in the higher wavelength region, the peaks around 1370 cm^−1^ and 1600 cm^−1^ are responsible for the D and G bands of the carbon fiber cloth (CFP), respectively^38^. The presence of MoSe_2_ on CC is confirmed by the relative strong Raman peak. The morphology and microstructure of the as-prepared MoSe_2_ nanosheets on CC were further studied by TEM observation. Figure 4(a) shows the typical TEM images of MoSe_2_ nanosheets. It is clearly observed that the exfoliated material consists of a very thin layer with a smooth surface. Corresponding high-resolution TEM (HRTEM) lattice fringes as shown in Fig. 4(b) demonstrate the single crystalline nature of the nanosheets. The HRTEM image reveals expected hexagonal lattice fringes with a lattice spacing of 0.28 nm, which is consistent with the lattice spacing of MoSe_2_ (1 0 0) planes.

**Figure 5:**
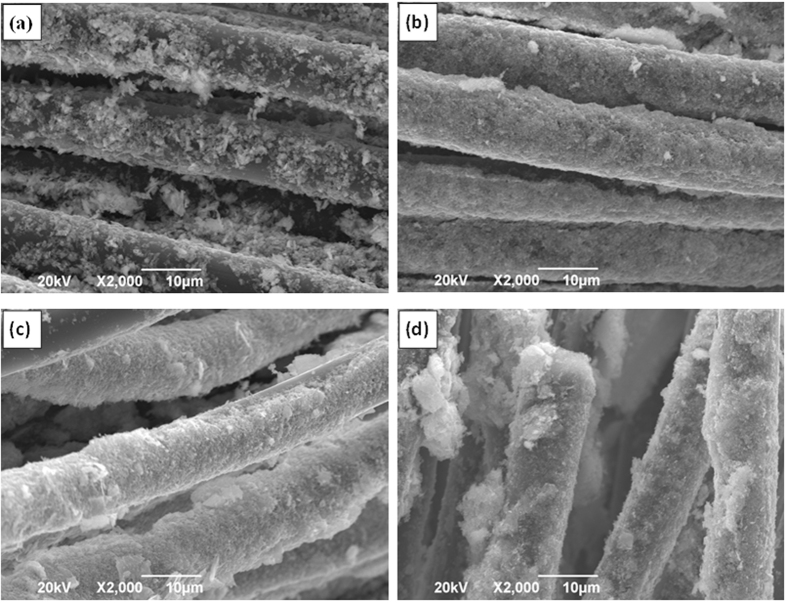
SEM images of MoSe_2_ nanosheets/carbon cloth (MoSe_2_/CC) prepared with different EPD time: (**a**) 30 min (MoSe_2_/CC-30), (**b**) 60 min (MoSe_2_/CC-60), (**c**) 120 min (MoSe_2_/CC-120), and (**d**) 180 min (MoSe_2_/CC-180).

As an advantage of the EPD method, the coverage and thickness of MoSe_2_ film on CC could be easily controlled by the deposition time. Therefore, a series of MoSe_2_/CC electrodes were prepared with different EPD times for the evaluation and optimization for HER performance. For the sake of discussion, the four samples prepared with different EPD time (30 min, 60 min, 120 min, 180 min) are labeled as MoSe_2_/CC-30, MoSe_2_/CC-60, MoSe_2_/CC-120, and MoSe_2_/CC-180, respectively. The SEM images (a–d) in Fig. 5 show the morphologies of these four samples, respectively. From a general view, the surfaces of all the carbon fibers in these four samples were all uniformly covered with the MoSe_2_ nanosheets film. However, in detail, the deposition of MoSe_2_ nanosheets in MoSe_2_/CC-30 is less than those of the other three, and some area of the fibers in MoSe_2_/CC-30 were still uncovered. It suggests that 30 min is relatively short for the full deposition of MoSe_2_ nanosheets on the entire area of each fiber in CC. In the other hand, the other three samples which undergoing more than 30 min EPD process also have some differences. According to the SEM images of pure CC in Fig. 2a, the statistical average diameter of smooth fibers in bare CC is about 9.67 μm, so the deposition thickness of MoSe_2_ nanosheets film coating on fibers can be calculated by minus the pristine fiber’s diameter. Based on the statistical calculation, the deposition thickness of these three electrodes (MoSe_2_/CC-60, MoSe_2_/CC-120 and MoSe_2_/CC-180) increased gradually with time, and the MoSe_2_/CC-180 contains the thickest MoSe_2_ nanosheets film reaching 3.2 μm (detail thicknesses are summarized in Table 1).

**Figure 6:**
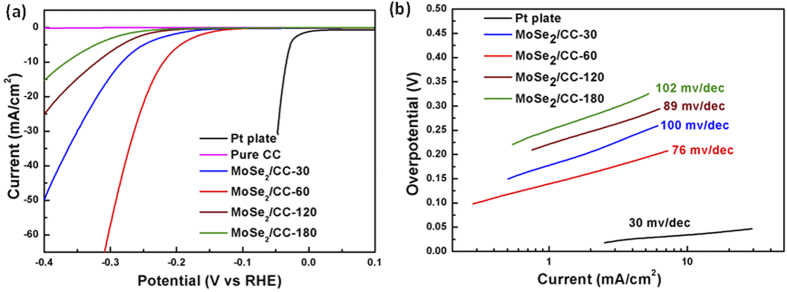
(**a**) Polarization curves obtained with pure carbon cloth, MoSe_2_/CC-30, MoSe_2_/CC-60, MoSe_2_/CC-120, MoSe_2_/CC-180 and commercial Pt plate; (**b**) the corresponding Tafel plots of MoSe_2_/CC-30, MoSe_2_/CC-60, MoSe_2_/CC-120, MoSe_2_/CC-180 and commercial Pt plate.

For MoSe_2_ materials, the basal edges have been identified as the active sites for the HER^16^. In this work, the uniform MoSe_2_ nanosheets film on a rough and curved surface is expected to not only maximize the exposed active sites but also overcome the limited electron/proton transport in HER. To evaluate the HER performance of the 3D flexible MoSe_2_/CC electrodes and influences of film thickness on HER performance, the samples prepared by various EPD times were directly applied as hydrogen evolution cathodes in 0.5 M H_2_SO_4_ solution using a three-electrode setup. Bare CC and commercial Pt plate were also tested as a comparison. A resistance test was made and iR compensation was applied for all the electrochemical measurements. Figure 6a shows the polarization curves in 0.5 M H_2_SO_4_ with a scan rate of 2 mV/s with current density normalized by geometric surface area (1 × 1 cm^2^) of these electrodes. Commercial Pt plate catalyst exhibits expected HER activity with a near zero over-potential. Although bare CC shows negligible HER activity, the four MoSe_2_/CC electrodes were all highly active toward the HER over this range of electrode potentials, implying the high activity of these electrodes arises from MoSe_2_ nanosheets. Among the four MoSe_2_/CC electrodes, the cathodic polarization curve recorded for the MoSe_2_/CC-60 displays the highest current density, 21 mA/cm^2^ at over-potential of around −0.25 V. Since the cathodic current density is proportional to the amount of evolved hydrogen, the large current density here indicates the prominent hydrogen evolution behavior of the MoSe_2_/CC-60 electrode. This may arise from the flexible electrode structure which brings in more active sites along with the optimized conductivity. Besides MoSe_2_/CC-60, the MoSe_2_/CC-30 held a higher current density and lower onset potential in contrast with MoSe_2_/CC-120 and MoSe_2_/CC-180.

In addition, Fig. 6b displays the Tafel plot and the fitting curves for the related electrodes. The Tafel equation η = a + b log j was used to obtain Tafel slope b, which is relative to the inherent property of the catalyst and indicates the rate-determining step in the whole HER process^19^. Besides, a smaller Tafel slope is preferred as it means a faster increase of hydrogen generation rate with increasing over-potential applied^11^. Commercial Pt plate is the most active material with a Tafel slope of 30 mV/decade in all these electrodes, and the linear part of the MoSe_2_/CC-60 Tafel plot under small over-potential is fitted to give a Tafel slope of 76 mV/decade, which is smaller than the Tafel slopes for pure MoSe_2_ electrode reported in the literatures^16^,^17^,^24^,^39^. Although MoSe_2_/CC-60 here has higher Tafel slope than commercial Pt plate, the 3D binder-free MoSe_2_/CC Electrodes still have a huge potential for practical HER application due to their low-cost and relative high electrochemical activity. The Tafel slopes of 100, 89 and 102 mV/decade were calculated for MoSe_2_/CC-30, MoSe_2_/CC-120, and MoSe_2_/CC-180, respectively. In the previous work the reported 120 mV/decade Tafel slope of MoSe_2_ on flat glassy carbon substrate indicates that the rate determining step was the discharge step, with a very small surface coverage of adsorbed hydrogen^24^. By extrapolation of the Tafel plots, exchange current densities for theses samples were also obtained, MoSe_2_/CC-60 exhibit the largest exchange current densities, which further revealing the excellent activity of the MoSe_2_/CC-60 catalysts toward HER. Based on these HER activity features of samples, it seems that the free energy barrier of the discharge step is reduced to be comparable with that of the following desorption or combination step, resulting in smaller Tafel slope of all MoSe_2_/CC electrodes. The roughness and surface curvature of CC may be able to expand or squeeze the active layers and thus change the electronic properties of the nano-films, which can tune the reaction barriers effectively.

**Figure 7:**
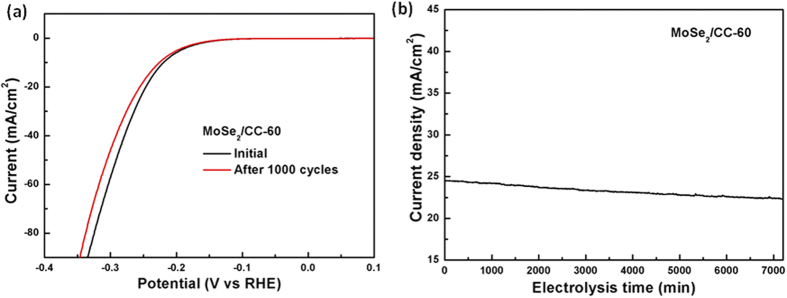
(**a**) The polarization curves of the MoSe_2_/CC-60 before and after 1,000 cycles of continuous operation, showing the catalyst structures have excellent electrochemical stability; (**b**) Time dependence of the current density of MoSe_2_/CC-60 under a static over-potential of 200 mV.

Stability is another important property of the HER electrocatalyst. To probe the durability of this flexible catalytic electrode in acidic environment, long-term potential cycling was performed by taking continuous cyclic voltammograms at an accelerated scanning rate of 100 mV/s for 1000 cycles. As shown in Fig. 7a, the polarization curve of the MoSe2/CC-60 electrode after 1000 cycles overlays almost exactly with the initial one, confirming the catalyst is highly stable to withstand accelerated degradation. Furthermore, the practical operation of the catalyst was examined by electrolysis at fixed potentials over extended periods. At over-potential of 200 mV (Fig. 7b), the catalyst current density remained stable at ∼23 mA/cm^2^ for electrolysis over 100 h with less than 10% loss in cathodic current density. This exceptional durability shows promise for practical applications of the catalysts over the long term.

**Figure 8:**
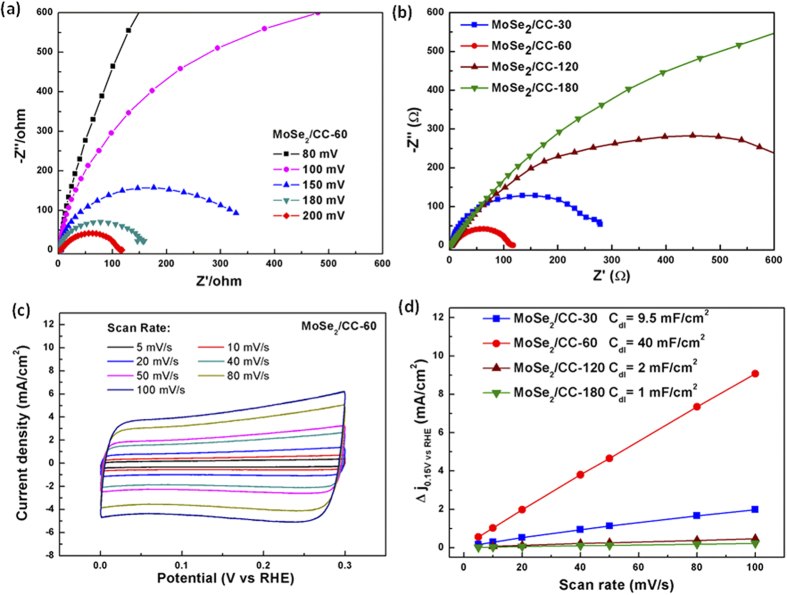
(**a**) Electrochemical impedance spectra of MoSe_2_/CC-60 at various HER overpotentials in 0.5 M H_2_SO_4_ (**b**) Electrochemical impedance spectra of MoSe_2_/CC-30, MoSe_2_/CC-60, MoSe_2_/CC-120 and MoSe_2_/CC-180. (**c**) Electrochemical cyclic voltammogram of MoSe_2_/CC-60 at different potential scanning rates. The selected potential range where no faradic current was observed is 0–0.3 V vs. RHE. (**d**) Linear fitting of the capacitive current differences of the catalysts against scan rates, the related calculated double layer capacitances of the four MoSe_2_/CC electrodes are also shown.

Based on the statistical calculation of the thickness of MoSe_2_ nanosheets films in different samples, the detail parameters of HER activities for these different samples were summarized in Table 1. The over-potential and Tafel slope of MoSe_2_/CC-60, MoSe2/CC-120 and MoSe2/CC-180 rose with the increase of thickness, and the exchange current densities of these three samples decreased with the enhancement of thickness. Moreover, the MoSe_2_/CC-30 showed a relatively lower on-set potential as well as a medium exchange current density, but a poor Tafel slope. It indicates that HER activities performances would be enhanced with the coverage of MoSe_2_ nanosheets film but also could be limited when the thickness of film was too large. After the coverage of MoSe_2_ nanosheets on CC to a certain degree, charge transfer kinetics under HER operating conditions for these samples would be blocked. To verify this, tests of electrochemical impedance spectroscopy (EIS) were conducted for these different MoSe_2_/CC electrodes to probe the electron-transfer kinetics involved. Figure 8(b) shows the Nyquist plots for catalysts at 200 mV over-potential with the corresponding equivalent circuit models insert, the related values equivalent circuit are listed in Table S1. As shown in Fig. 8(b), the observed semicircle is mainly attributed to the charge transfer resistance (R_ct_) of H^+^ reduction at the electrode-electrolyte interface. The MoSe_2_/CC-30 and MoSe_2_/CC-60 showed a much less charge transfer resistance than MoSe2/CC-120 and MoSe_2_/CC-180. This demonstrates that the thickness of MoSe_2_ nanosheets film greater than about 1 μm dramatically decreases the electron transfer. And the MoSe_2_/CC-60 displays a better charge transfer than MoSe_2_/CC-30, which may due to the more MoSe_2_ nanosheets on CC and more active site for H^+^ reduction at the electrode-electrolyte interface. What’s more, the charge transfer resistance (Rct) of MoSe_2_/CC-60 diminished markedly with increasing over-potential from −80 mV to −200 mV, suggesting improved electron-transfer kinetics with increasing over-potentials (Fig. 8(a)). In addition, the small solution resistances (Rs) of all the samples benefiting from the high conductivity of CC are favorable for the practical applications.

Besides the charge transfer kinetics, electrodes’ electrochemically active surface areas which related with the effective active area in HER would also be affected by the coverage and orientation of MoSe_2_ nanosheets film in the MoSe_2_/CC electrodes here. To estimate the effective surface areas of our MoSe_2_-based catalysts, electrochemical double-layer capacitances (EDLCs), Cdl, were measured by a simple cyclic voltammetry method. Within the potential range without faradic current, slow voltage scan rates were used for the accurate measurement of EDLCs. The halves of the positive and negative current density differences of the four MoSe_2_/CC electrodes at the center of the scanning potential ranges are plotted versus the variable voltage scan rates in Fig. 8(d), in which the slopes are the EDLCs. As shown in Fig. 8d, MoSe_2_/CC-60 exhibits the Cdl value of 40 mF, greatly larger than MoSe_2_/CC-30 (9.5 mF), MoSe_2_/CC-120 (2 mF), and MoSe_2_/CC-180 (1 mF). This result reveals that MoSe_2_/CC-60 has the largest effective active area among the MoSe_2_-based catalysts, which contributes to its superior HER activity. In view of the active surface areas results, the optimized coverage of MoSe_2_ nanosheets film in the MoSe_2_/CC electrodes by EPD would help the electrode to expose more active area for HER which would also benefit the practical application of MoSe_2_-based HER electrodes.

## Conclusion

In conclusion, we successfully prepared the MoSe_2_ nanosheets on the carbon fiber surface of carbon cloth by a simple electrophoretic deposition method. The resulting products exhibited superior HER activity and stability. The unique structure of the MoSe_2_/CC enhanced the exposed active sites of nanosheets and improved electron transfer between the catalysts and the electrode. The present work proposed an effective route to increase the catalytic active sites and the electrical conductivity of metal dichalcogenides-based HER electrodes, enabling their potential to replace Pt as an electrocatalyst in HER.

## Experimental Section

### Preparation of MoSe_2_ nanosheets

MoSe_2_ powder was firstly exfoliated to nanosheets by hydrothermal intercalation and exfoliation method^37^. Typically, preparation 10 g/L LiOH solution, the solvent is ethylene glycol and the solute is LiOH. Then, the bulk MoSe_2_ powder was added to the above solution. The mixture solution was transferred into 50 ml Teflon-lined autoclave, sealed tightly, and heated at 220 °C for 24 h. Colloidal suspensions of MoSe_2_ nanosheets can be readily prepared by suspending the lithiated MoSe_2_ powder in deionized water.

### Preparation of MoSe_2_/CC electrodes

The EPD method was used to prepare MoSe_2_/CC electrodes. In a typical process, 0.1 g MoSe_2_ nanosheets was firstly dispersed in 100 ml solution (ethyl alcohol: de-ionized water = 1:1) and sonicated for 1 h at room temperature. A uniform and stable colloid of MoSe_2_ nanosheets was obtained. A stainless steel substrate and CC were used as the positive electrode and the negative electrode, respectively. The electrodes were vertically oriented and separated by 1 cm in a beaker containing the MoSe_2_ colloid. A direct-current voltage of 100 V was applied to the suspension between the two opposite electrodes with deposition times ranging from 30 to 180 min. After the deposition on CC, samples were dried at 60 °C for 12 h, and then dried at 150 °C for 1 h to improve the contract.

### Characterization

The SEM images were taken with JEOL JSM-6360. The microstructures of MoSe_2_ nanosheets were investigated using transmission electron microscopy (TEM, JEOL JEM-3010). The XRD data were acquired by Kratos Analytical Ltd using Cu Ka source. Raman spectra were measured with the InVia Raman microscope at excitation laser wavelength of 532 nm.

### Electrochemical studies

Three electrodes configuration were employed, and all the electrochemical measurement was carried by employing a CHI 660D workstation at ambient temperature. The working electrode was the MoSe_2_/CC, the reference electrode was saturated calomel electrode and the counter electrode was a graphite rod. Liner sweep voltammetry with scan rate of 2 mV/s and cyclic voltammetry with different scan rates were conducted in 0.5 M H_2_SO_4_. In all measurements, we used saturated calomel electrode (SCE) as the reference. The potential was converted to the RHE reference electrode by E (vs RHE) = E (vs SCE) + E_SCE_ + 0.0591 pH.

## Additional Information

**How to cite this article**: Liu, Y. *et al.* 3D Binder-free MoSe_2_ Nanosheets/Carbon Cloth Electrodes for Efficient and Stable Hydrogen Evolution Prepared by Simple Electrophoresis Deposition Strategy. *Sci. Rep.* **6**, 22516; doi: 10.1038/srep22516 (2016).

## Supplementary Material

Supplementary Information

## Figures and Tables

**Table 1:** The related HER parameters of various electrodes.

Catalyst	Thickness(μm)	Tafel slope(mV/decade)	Current density(j, mA/cm^2^)^a^	Overpotential at 10mA/cm^2^ (V)	Exchange currentdesity (j_0_, mA/cm^2^)^b^	Ref.
MoSe_2_ film	–	105–120	–	–	2.0 × 10^−3^	24
MoSe_2_ nanosheets/glassy carbon	–	101	–	0.29	–	16
MoSe_2_/CC-30	0.15	100	1.8	0.282	9.75 ×10^−3^	This work
MoSe_2_/CC-60	0.81	76	6.0	0.220	1.53 × 10^−2^	This work
MoSe_2_/CC-120	1.3	89	0.62	0.321	3.68 × 10^−3^	This work
MoSe_2_/CC-180	3.2	102	0.39	0.366	2.32 × 10^−3^	This work

^a^Cathodic current densities (j) were recorded at η = 200 mV.

^b^The exchange Current densithy (j_0_, mA/cm^2^) were obtained by extrapolation of the Tafel lines.
