# Supplementary material for: 3D Binder-free MoSe2 Nanosheets/Carbon Cloth Electrodes for Efficient and Stable Hydrogen Evolution Prepared by Simple Electrophoresis Deposition Strategy
Source: Sci Rep. 2016 Mar 7;6:22516. doi: 10.1038/srep22516 (PMC4780084; doi:10.1038/srep22516)
Supplement: Supplementary Information [file srep22516-s1.doc]

**Supplementary Information**

**For**

**3D Binder-free MoSe_2_ Nanosheets/Carbon Cloth Electrodes for Efficient and Stable Hydrogen Evolution Prepared by Simple Electrophoresis Deposition Strategy**

Yundan Liu ^a,b^, Long Ren ^a,b,c^, Zhen Zhang ^a,b^, Xiang Qi ^a,b^*, Hongxing Li ^a,b^,

Jianxin Zhong ^a,b^*

*^a^ Hunan Key Laboratory of Micro-Nano Energy Materials and Devices, Xiangtan University, Hunan 411105, PR China*

*^b^ Laboratory for Quantum Engineering and Micro-Nano Energy Technology and School of Physics and Optoelectronics, Xiangtan University, Hunan 411105, PR China*

*^c^ Institute for Superconducting and Electronic Materials, Australian Institute for Innovative Materials, University of Wollongong, Innovation Campus, North Wollongong, New South Wales 2500, Australia*

^*^ Corresponding author: School of Physics and Optoelectronics, Xiangtan University, Hunan 411105, P. R. China

**E-mail address:** [**xqi@xtu.edu.cn**](mailto:xqi@xtu.edu.cn) **or** [**jxzhong@xtu.edu.cn**](mailto:jxzhong@xtu.edu.cn)

**Additional Figures**

**Figure captions**

Figure S1. X-ray diffraction pattern of the bulk MoSe_2_ and the as-prepared MoSe_2_ nanosheets prepared by hydrothermal intercalation and exfoliation method.

Figure S2. Raman spectra of pure carbon cloth.

Figure S3. Electrochemical cyclic voltammogram of MoSe_2_/CC-30, MoSe_2_/CC-120, MoSe_2_/CC-180 at different potential scanning rates. The selected potential range where no faradic current was observed is 0 - 0.3 V vs. RHE.

Table S1. The data from fitting the impedance data in Figure 8 (b) to an equivalent circuit.

**Figure S1**

**
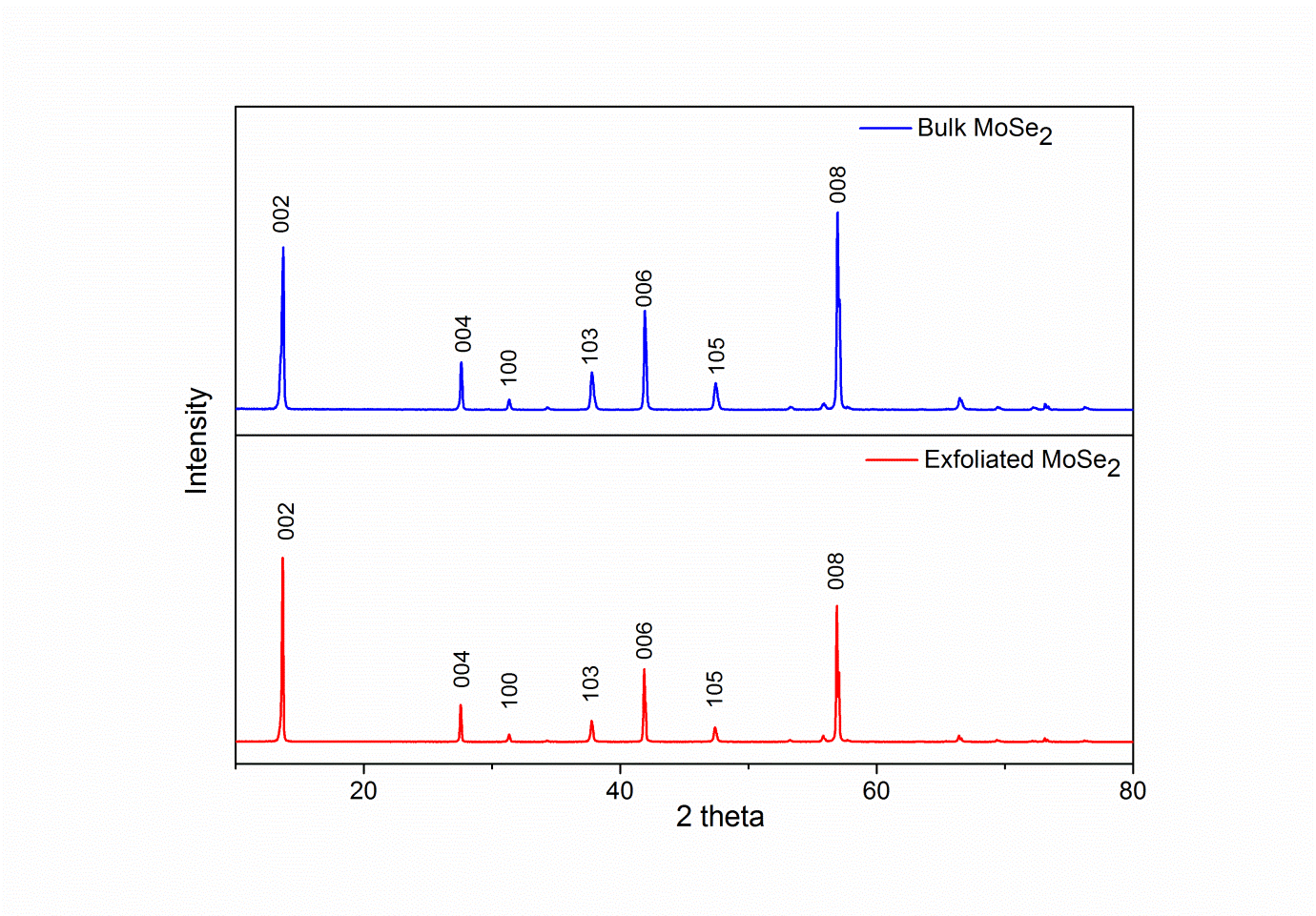
**

**Figure S2**

**
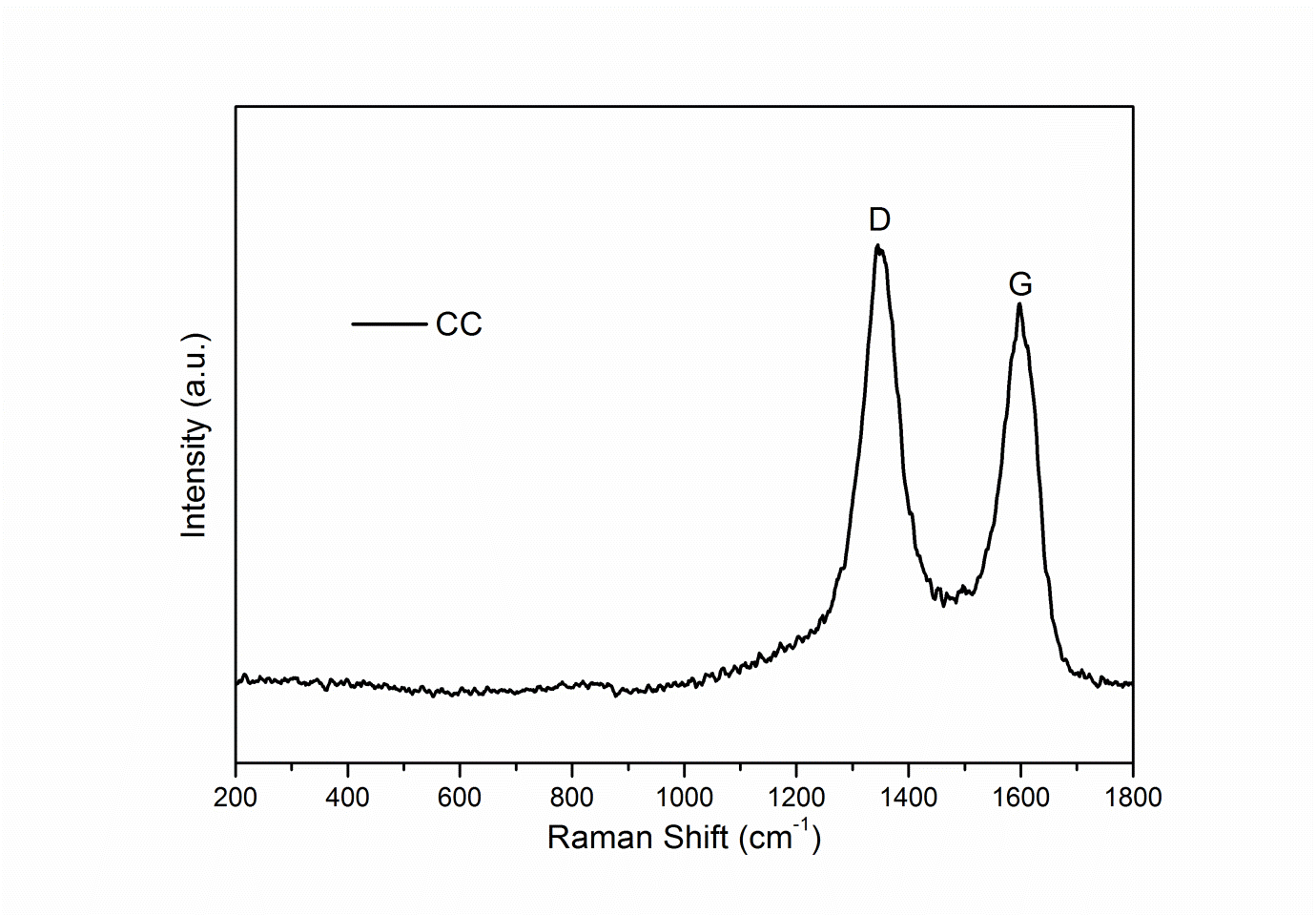
**

**Figure S3.**

**(a)**

**
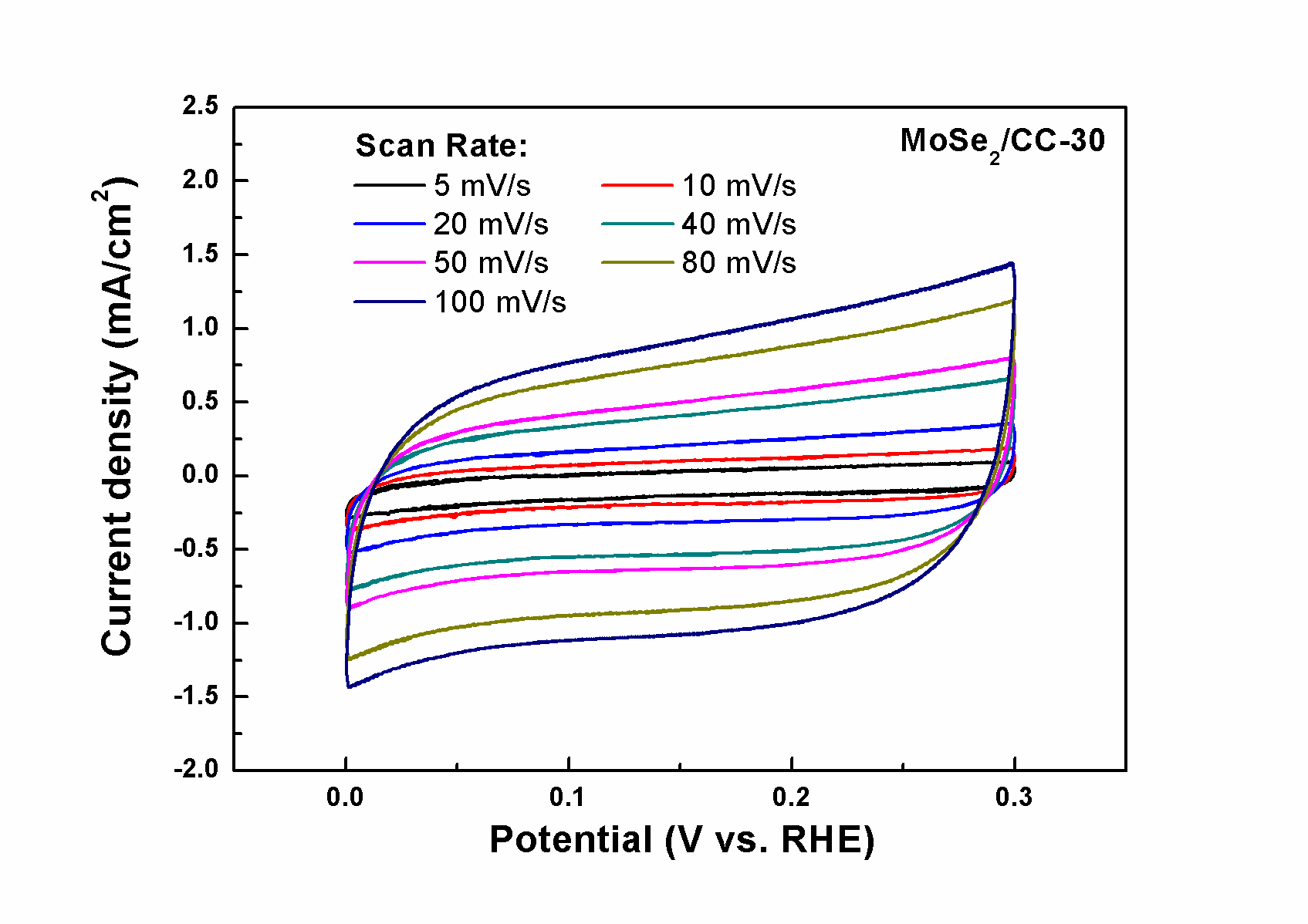

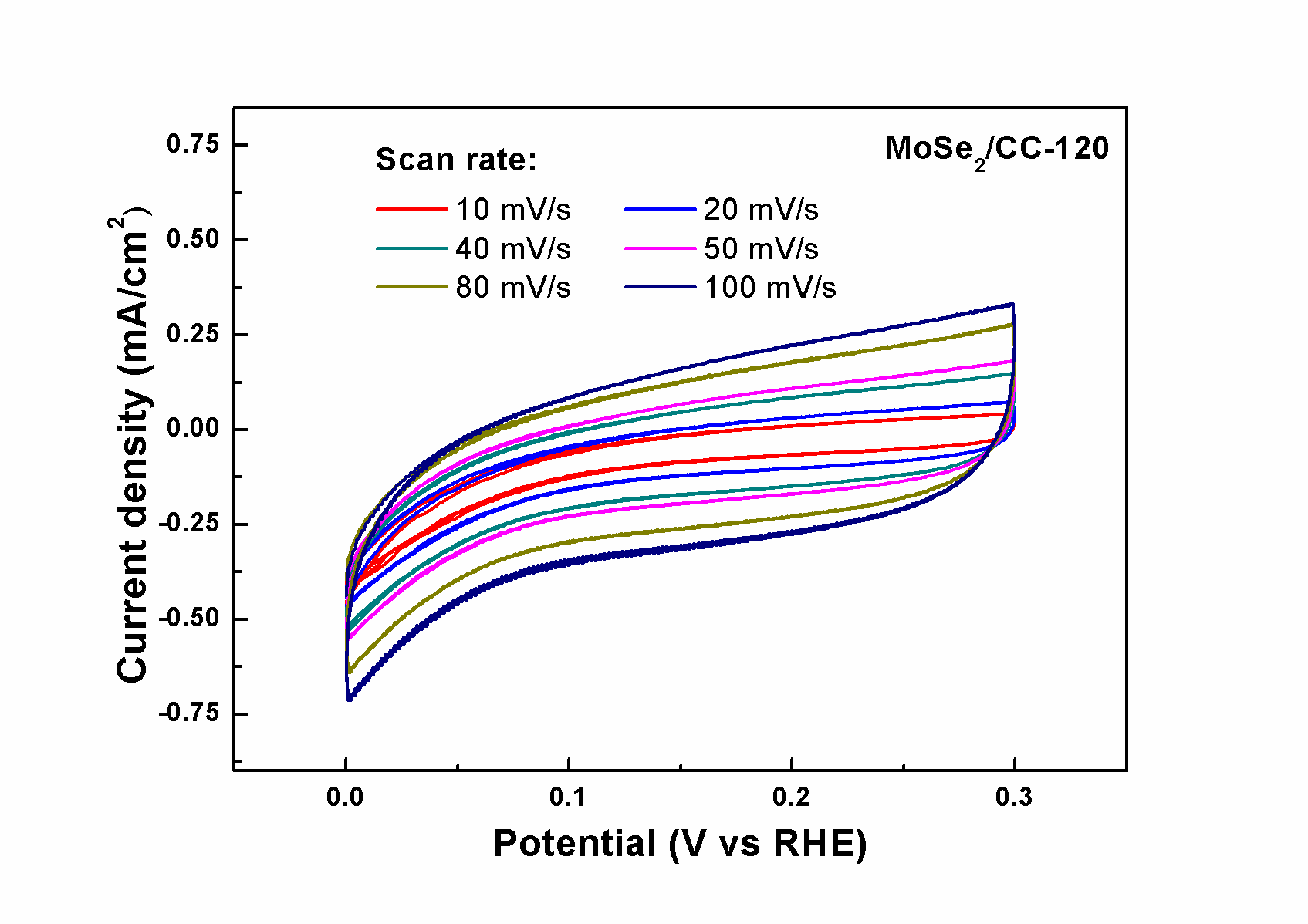

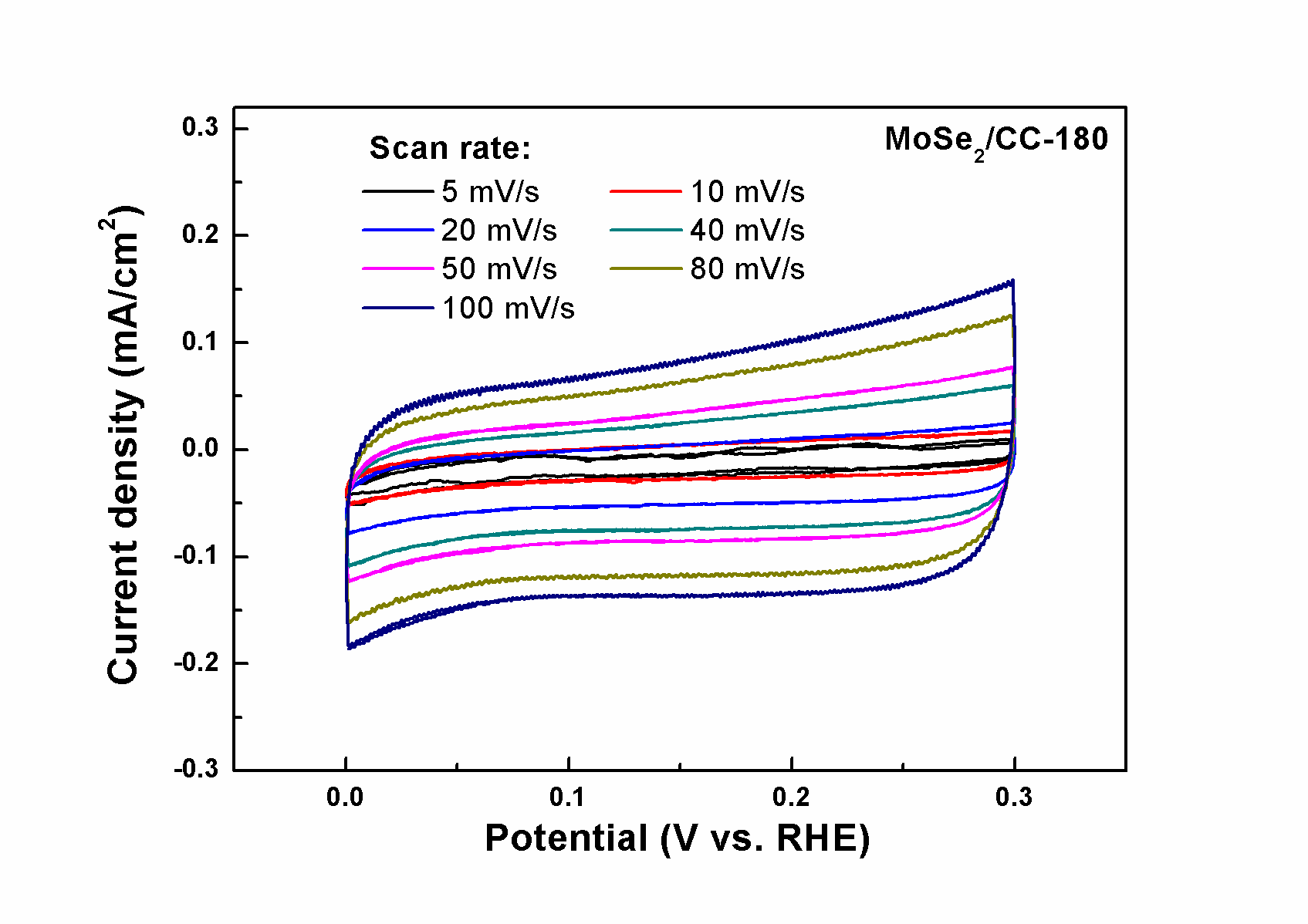
**

**(b)**

**(c)**

**Table S1.**

| Catalyst | Solution resistance  (Rs, Ω/cm^2^) | Charge transfer resistance  (Rct, Ω/cm^2^) | Constant phase element  (CPE, µF) |
| --- | --- | --- | --- |
| MoSe_2_/CC-30 | 5.266 | 284.9 | 8590 |
| MoSe_2_/CC-60 | 4.069 | 116.1 | 2150 |
| MoSe_2_/CC-120 | 3.756 | 887.5 | 202 |
| MoSe_2_/CC-180 | 3.161 | 2347 | 235 |
